# Histopathological Finding of Microdamage Accumulation in Atypical Subtrochanteric Femoral Fracture

**DOI:** 10.1155/2021/6624414

**Published:** 2021-03-20

**Authors:** Yo Watanabe, Naoki Kondo, Tomomi Fukuhara, Norio Imai, Masahiko Yamada, Naoto Endo

**Affiliations:** ^1^Division of Orthopedic Surgery, Department of Regenerative and Transplant Medicine, Niigata University Graduate School of Medical and Dental Sciences, 1-757 Asahimachi-dori, Chuo-Ku, Niigata 951-8510, Japan; ^2^Division of Comprehensive Musculoskeletal Medicine, Niigata University Graduate School of Medical and Dental Sciences (Orthopedic Surgery), 1-757 Asahimachi-dori, Chuo-Ku, Niigata 951-8510, Japan; ^3^Division of Orthopedic Surgery, Niigata Prefectural Tsubame Rosai Hospital, 633 Sawatari, Ooaza, Tsubame, Niigata 959-1228, Japan

## Abstract

Atypical femoral fracture is a low-energy stress fracture in the subtrochanteric region or the femoral shaft and is a complication of the long-term use of bisphosphonates. Histopathological findings of atypical femoral fractures have not been clarified. Herein, we report the case of a 61-year-old woman who fell while walking, which prompted her to visit our facility. She had a 7-year history of alendronate use to treat osteoporosis. A radiograph showed an atypical subtrochanteric femoral fracture, following which she underwent a primary surgery, where an intramedullary femoral nail was used. Implant breakage was discovered 8 weeks after the primary surgery. The patient underwent a revision surgery in which the entry point for the revised intramedullary hole was created to prevent varus position. The lag screw was successfully inserted into the center of the femoral head. Cancellous bone, isolated from the right ilium, was autogenously implanted into the fracture site. Fracture healing was promoted using low-intensity pulse ultrasonography. Callus formation was detected on a radiograph, and full weight-bearing was advised 12 weeks after the revision surgery. The fracture had healed completely at 13 months after the revision surgery. The patient was able to walk without support and could independently perform activities of daily life. Laboratory findings suggested that the concentrations of her bone formation markers were normal, while those of bone resorption markers were elevated. Iliac bone histomorphometry did not reveal severely suppressed bone turnover. In the cortex of fracture site, the lacunar density was markedly lower than the osteocyte density, and microcracks were detected, suggesting impaired osteocyte function and a low potential for fracture healing. This case is notable because it helps to clarify the histopathological findings of atypical femoral fractures.

## 1. Introduction

Atypical femoral fracture (AFF), a low-energy stress fracture sustained in the subtrochanteric region of the femoral shaft, is a complication of the long-term use of bisphosphonates (BPs). BPs inhibit bone resorption and formation. Recently, many cases of AFF caused by the application of low energy have been reported [1–3]. Severely suppressed bone turnover (SSBT) is also reported as one of the main causes of AFF [1, 4, 5]. Odvina et al. have reported 9 cases of biopsy-proven SSBT. In these patients, iliac bone biopsy has revealed a remarkably low bone turnover based on bone histomorphometry [5]. However, the exact mechanism underlying the pathophysiology of AFF remains unknown.

Herein, we present our experience of treating a patient on a regimen of BP who sustained an atypical femoral subtrochanteric fracture and in whom implant breakage occurred postoperatively. In this patient, we conducted histopathological examination of both the iliac bone and the fracture site, including an analysis of microcracks and osteocyte density.

## 2. Case Presentation

A 61-year-old woman fell while walking, which prompted her to visit our facility. She was previously diagnosed with mammary carcinoma, osteoporosis, and hypertension. She had been taking alendronate (35 mg/week) for 7 years to treat osteoporosis.

**Figure 1:**
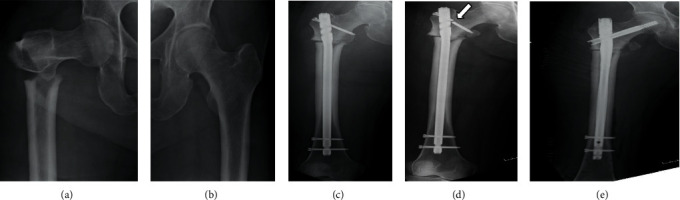
Radiographs of both femurs. The initial right femoral plain radiograph shows an atypical subtrochanteric fracture (a). On the contralateral side, no fracture is detected at the subtrochanteric site (b). The intramedullary nail is inserted during primary surgery with osteosynthesis (c). The intertrochanteric screw is broken (white arrow) (d), and the fracture site is more displaced than that shown in (c). Revision surgery is then performed. The intramedullary nail is reinserted, and the lag screw is inserted in the femoral neck and head (e).

A radiograph showed a right atypical femoral subtrochanteric fracture (Figure 1(a)), which fulfilled the criteria for AFF, established by the American Society for Bone and Mineral Research (ASBMR) task force [6]. No fracture was found on the lateral cortex of the contralateral side (left) of the femur (Figure 1(b)). A primary surgery was then performed, and an intramedullary femoral nail was used (Figure 1(c)). After the primary surgery, the patient was advised nonweight-bearing activities for a period of 2 weeks.

**Figure 2:**
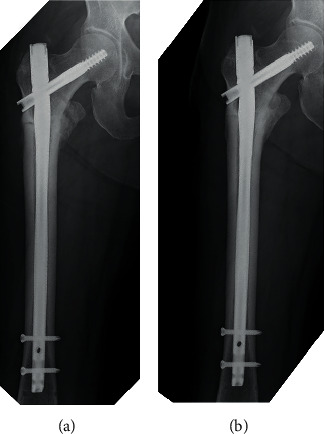
Time course of radiographs after the revision surgery. Three months after the revision surgery, callus formation is detected at the fracture site (a). At 13 months after the revision surgery, the fracture site has healed (b).

At postoperative 3-week follow-up, one-third partial weight-bearing was advised, which progressed to full weight-bearing at postoperative 6 weeks. However, implant breakage was detected at postoperative 8 weeks (Figure 1(d)), and a revision surgery was subsequently performed. The entry point for the revised intramedullary hole was created to prevent varus position, and good anatomical reduction was achieved (Figure 1(e)). The lag screw was successfully inserted into the center of the femoral head. Additionally, the cancellous bone isolated from the right ilium was autogenously implanted into the fracture site. One day after the revision surgery, one-third partial weight-bearing was advised. Low-intensity pulse ultrasonography was also performed to promote fracture healing. Because the patient had breast cancer, she was not prescribed teriparatide, a parathyroid hormone (PTH) agent. At 12 weeks after the revision surgery, full weight-bearing was advised because callus formation was detected on follow-up radiographs (Figure 2(a)). Finally, the fracture healed by 13 months after the revision surgery (Figure 2(b)), and the patient could walk without support and independently perform activities of daily life.

The laboratory findings after the primary surgery are shown in Table 1. The concentrations of serum calcium and inorganic phosphorus were within the normal range. The serum concentration of 25-hydroxyvitamin D was 26 ng/mL, which was slightly lower than the reference value (>30 ng/mL). The concentration of PTH (high-sensitivity assay) was 560 pg/mL, which was slightly higher than the reference range (160–520 pg/mL), as was the tartrate-resistant acid phosphatase 5b (TRACP-5b) concentration (446 mU/dL; reference range: 120–420 mU/dL). Her urine N-terminal telopeptide (uNTx) concentration of 113.3 nmol BCE/mmol-Cr was higher than the reference range (14.3–89.0 nmol BCE/mmol-Cr), while her urine deoxypyridinoline/creatinine (FDPY/Cre) concentration of 20.0 nM/mMcre was remarkably higher than the reference range (2.8–7.6 nM/mMcre). These findings suggest that the concentrations of her bone formation markers were within the normal range, whereas the concentration of her bone resorption markers was elevated.

**Figure 3:**
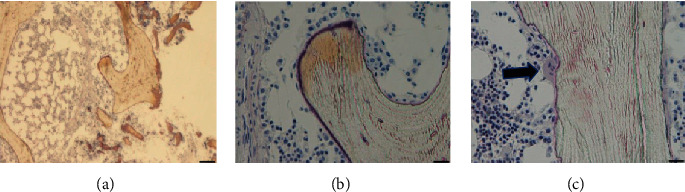
Bone staining (Villanueva bone staining) of the biopsied iliac bone. Trabecular bone (a) ×100, osteoid tissue (b) ×200, and osteoclasts (black arrow) (c) × 200 are detected. Bar: (a) 100 *μ*m; (b) and (c) 50 *μ*m.

Histopathological findings of ilium biopsy specimens revealed a barely detectable, thin osteoid. Osteoclasts were detected around the trabecular bone (Figure 3). The bone histomorphometric findings of her right ilium were as follows: bone volume, 24.15%; trabecular thickness, 147.82 *μ*m; trabecular width, 32.4 *μ*m; osteoid volume per tissue volume, 0.09%; osteoid volume per bone volume, 0.38%; osteoid surface, 7.13%; osteoid thickness, 3.75 *μ*m; and osteoblast surface, 1.3%. For bone resorption parameters, the eroded surface was 5.56%; osteoclast surface, 0.4%; and fibrosis bone volume, 0% (Table 2). These data indicated that the bone volume parameters were within the normal range, the bone formation parameters (osteoid surface and osteoid thickness) were lower than the reference range, and the bone resorption parameters were within the normal range (Table 2).

**Figure 4:**
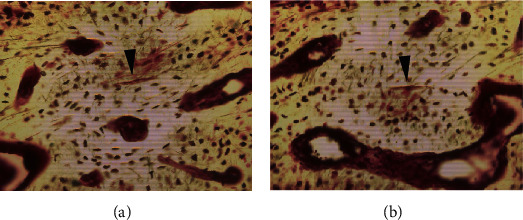
Microcracks are identified using basic fuchsin staining. Fuchsin-positive microcracks are detected (black arrowheads in (a, b) in the cortex).

The number of osteocytes and empty lacunae in the fracture site specimen (cortex) was measured. The osteocyte density (394.2 N/mm^2^) was remarkably higher than the empty lacunar density (8 N/mm^2^; Table 3). Seven microcracks were found in the cortex of the fracture site (Figure 4), with a crack density of 0.79 N/mm^2^ (Table 4).

Informed consent was obtained from the patient for the publication of this case report and the accompanying images. All procedures were conducted in accordance with the Declaration of Helsinki (1964).

## 3. Discussion

The present case is worth reporting in that a detailed histological examination of an atypical femoral subtrochanteric fracture case was performed to investigate both the bone metabolic status and condition of the fracture site. To the best of our knowledge, this is the first case in which both the osteocyte and lacunar density at the AFF site were evaluated. The patient was diagnosed with AFF because her case met all of the 5 major criteria described by the ASBMR task force [6].

Long-term use of BPs is correlated with the incidence of AFF. In a previous report, 94% of patients with AFF (291/310) were treated with BPs for longer than 5 years [7], while in another report, 78% of patients with AFF were treated with BPs [8]. In fact, the odds ratio for the incidence of AFF was 35 among patients taking BPs for less than 2 years, and it was 115 among those taking BPs for 5–9 years, which indicates that prolonged use of BPs is associated with a 3-fold higher risk of AFFs [9]. In the present case, the patient had been treated with alendronate for 7 years. However, the association between the incidence of AFF and BP treatment is not completely ruled out.

The association between AFF and biopsy-proven SSBT has also been reported [5]. Biopsy-proven SSBT is characterized by the disappearance of the osteoid surface and absence of cellular content, such as osteoblasts and osteoclasts, surrounding the trabecular bone [5]. We previously experienced a case of SSBT (a 36-year-old woman with bilateral atypical femoral subtrochanteric fractures) [1], but other reported cases of AFF were not related to SSBT [10, 11].

In the present case, both the osteoid thickness and osteoid surface were identified, and osteoclasts were found; therefore, no SSBT findings were confirmed. Bone histomorphometric findings demonstrated that bone volume and bone resorption parameters were within the normal range, while that of bone formation parameters were low, suggesting that the long-term use of alendronate did not inhibit bone resorption. In addition, the concentration of bone resorption markers, such as TRACP-5b, uNTx, and FDPY/Cre, was slightly above the normal range.

In the early phase of fracture healing, osteocytes undergo apoptosis and express cell apoptotic caspase-3 in the vicinity of the fracture site. The degree of osteocyte apoptosis can be monitored by measuring the empty lacunar density at the fracture site [12]. Using experimental animal models of fracture repair, 3 studies have examined the osteocyte number after fracture using histomorphometry [13–15]. Moreover, 2 further studies have revealed osteocyte apoptosis in the acute phase, in close proximity to the fracture [16, 17]. However, no study has reported the comparison between osteocyte density and empty lacunar density at the fracture site. In the present case, the empty lacunar density was markedly lower than the osteocyte density, suggesting that fracture healing was delayed.

In our case, histological examination based on basic fuchsin staining demonstrated that microcracks were present in the cortex of the fracture site (Figure 4). The microcrack density was higher than normal [18]. Vashishth et al. have reported that the decline in the osteocyte lacunar density of human cortical bone is associated with microcrack accumulation and an age-related increase in bone porosity [19], but this finding is not consistent with that of the present case because the lacunar density was not higher than the osteocyte density.

Iwata et al. have reported the case of a woman with atypical femoral subtrochanteric fractures who had been treated with zoledronate for 9 years for her bone metastasis of mammary carcinoma—the findings coincided with biopsy-proven SSBT and microdamage accumulation [4]. Similarly, microdamage accumulation was one of the causes of AFF in the present case.

Collectively, we consider that the low lacunar density and the existence of microcracks suggest delayed union or a decreased fracture healing ability. In the present case, because of implant failure, a revision surgery was required. Nonrigid fixation with a screw and a short time of nonweight-bearing were likely to lead to delayed fracture healing and breakage of the screw. The primary surgery did not lead to acceptable anatomical reduction; however, the revision surgery achieved acceptable anatomical reduction, and a lag screw was inserted into the right femoral head to achieve a more rigid fixation. Additionally, iliac bone grafting was performed after the fracture site was curetted to promote fracture healing. The duration of bone healing in AFF cases is generally longer than that in typical femoral fractures [20–22]. Additionally, the presence of an anterior gap, lateral gap, and cortical breakage at the fracture site, as shown in the postoperative radiographs, is associated with delayed union [22].

Despite its novelty, the findings of this case had some limitations: double tetracycline labeling was not performed; consequently, dynamic bone parameters could not be evaluated. Additionally, serum or urine pentosidine concentration was not measured; therefore, bone quality could not be evaluated.

## 4. Conclusion

We reported a case in which the patient had sustained an atypical femoral subtrochanteric fracture that presented with delayed union. This patient had been on alendronate therapy for 7 years. Although both the iliac bone and the fracture site were histologically evaluated, the iliac bone biopsy did not reveal SSBT. At the fracture site, the lacunar density was markedly lower than the osteocyte density, and microcracks were also detected, indicating a low potential for fracture healing and impaired osteocyte function.

## Figures and Tables

**Table 1:** Laboratory findings after the patient's primary surgery.

Items	Abbreviations	Units	Reference values	Present case findings
Serum calcium	sCa	mg/dL	8.7–10.0	9.9
Inorganic phosphorus	iP	mg/dL	2.5–4.6	4.2
Alkaline phosphatase	ALP	U/L	115–359	254
Bone-specific alkaline phosphatase	BAP	U/L	9.6–35.4	16.3
Tartrate-resistant acid phosphatase 5b	TRACP-5b	mU/dL	120–420	446
Intact parathyroid hormone	Intact PTH	pg/mL	10–65	34
Uncarboxylated osteocalcin	ucOC	ng/mL	<4.50	4.29
Urine type I collagen cross-linked N-telopeptide	uNTx	nmol BCE/mmol-Cr	14.3–89.0	113.3
Urine deoxypyridinoline/creatinine	FDPY/CRE	nmol/mmol-Cr	2.8–7.6	20.0
25-Hydroxyvitamin D	25-OH-D	ng/mL	≥30	26.0

BCE: bone collagen equivalent.

**Table 2:** Bone histomorphometric findings in the biopsied ilium specimen.

	Parameter	Abbreviations	Unit	[55-64F]	Present case
				Reference values [23]	
Bone volume	Bone volume	BV/TV	%	20.79 ± 4.37	24.15
	Trabecular thickness	Tb.Th	*μ*m	133.0 ± 34.4	147.82
	Trabecular width	W.Th	*μ*m	30.34 ± 3.45	32.4
Bone formation	Osteoid volume (tissue volume)	OV/TV	%	0.44 ± 0.24	0.09
	Osteoid volume (bone volume)	OV/BV	%	2.17 ± 1.14	0.38
	Osteoid surface	OS/BS	%	16.7 ± 6.99	7.13
	Osteoid thickness	O.Th	*μ*m	9.16 ± 1.94	3.75
	Osteoblast surface	Ob.S/BS	%	6.05 ± 3.83	1.3
Bone resorption	Eroded surface	ES/BS	%	4.14 ± 2.12	5.56
	Osteoclast surface	Oc.S/BS	%	0.82 ± 0.80	0.4
	Fibrous bone volume	Fb.V/TV	%	0	0

**Table 3:** Osteocyte density and empty lacunar density at the fracture site.

Parameter	Present case	Unit
Bone tissue volume	5.14	mm^2^
The number of osteocytes	2026	N
Osteocyte density	394.2	N/mm^2^
The number of empty lacuna	41	N
Empty lacuna density	8	N/mm^2^

**Table 4:** Measurement of microcracks at the fracture site.

Parameter	Abbreviations	Present case	Unit	Reference data [18]	
Bone area	B.Ar	8.84	mm^2^		
The number of microcracks	Cr.N	7	N		
Total crack length	Cr.S	615.66	*μ*m		
Average crack length	Cr. Le.	87.94	*μ*m		
Crack density	Cr. Dn	0.79	N/mm^2^	0.21	N/mm^2^
Crack surface density	Cr.S.Dn	69.64	*μ*m/mm^2^	19.5	*μ*m/mm^2^

## Data Availability

The clinical details, radiological and histopathological details, and investigation reports used to support the findings of this case report are available from the corresponding author upon request.
